# Strategies for monitoring and combating resistance to combination kinase inhibitors for cancer therapy

**DOI:** 10.1186/s13073-017-0431-3

**Published:** 2017-04-21

**Authors:** Leanne G. Ahronian, Ryan B. Corcoran

**Affiliations:** 1 Massachusetts General Hospital Cancer Center, Boston, MA 02129 USA; 2 Department of Medicine, Harvard Medical School, Boston, MA 02115 USA

## Abstract

Targeted therapies such as kinase inhibitors and monoclonal antibodies have dramatically altered cancer care in recent decades. Although these targeted therapies have improved patient outcomes in several cancer types, resistance ultimately develops to these agents. One potential strategy proposed to overcome acquired resistance involves taking repeat tumor biopsies at the time of disease progression, to identify the specific molecular mechanism driving resistance in an individual patient and to select a new agent or combination of agents capable of surmounting that specific resistance mechanism. However, recent studies sampling multiple metastatic lesions upon acquired resistance, or employing “liquid biopsy” analyses of circulating tumor DNA, have revealed that multiple, heterogeneous resistance mechanisms can emerge in distinct tumor subclones in the same patient. This heterogeneity represents a major clinical challenge for devising therapeutic strategies to overcome resistance. In many cancers, multiple drug resistance mechanisms often converge to reactivate the original pathway targeted by the drug. This convergent evolution creates an opportunity to target a common signaling node to overcome resistance. Furthermore, integration of liquid biopsy approaches into clinical practice may allow real-time monitoring of emerging resistance alterations, allowing intervention prior to standard detection of radiographic progression. In this review, we discuss recent advances in understanding tumor heterogeneity and resistance to targeted therapies, focusing on combination kinase inhibitors, and we discuss approaches to address these issues in the clinic.

## Background

**Fig. 1:**
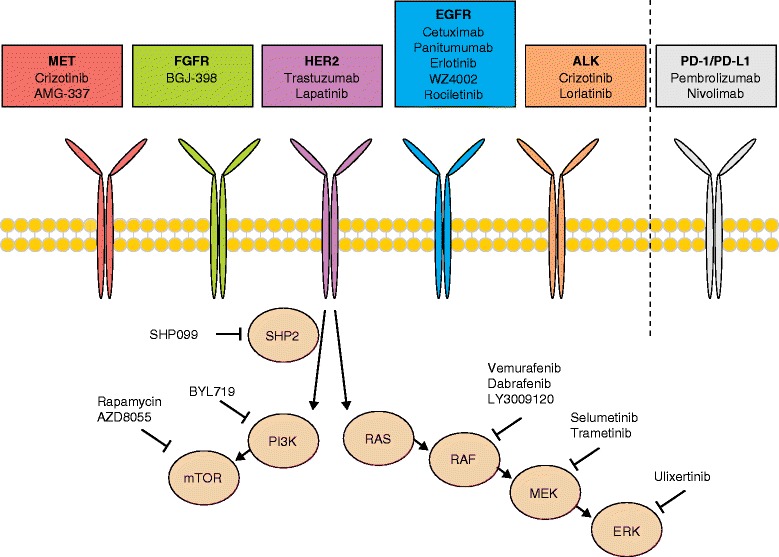
Agents used for targeted cancer therapy. This figure details the agents discussed in this review, including monoclonal antibodies and kinase inhibitors targeting multiple receptors, including MET, FGFR (fibroblast growth factor receptor), HER2 (human epidermal growth factor receptor 2), EGFR (epidermal growth factor receptor), and ALK (anaplastic lymphoma kinase). Additionally, kinase and phosphatase inhibitors targeting downstream effectors of these receptors are indicated, including SHP2 and members of the PI3K (phosphatidylinositol-3-kinase) and MAPK (mitogen-activated protein kinase) pathways. Lastly, monoclonal antibodies targeting receptors regulating immune response, PD-1 and PD-L1, are also discussed

In the past decade, genetic information gathered from patient tumors has revolutionized approaches to the use of targeted therapies in cancer care. These personalized treatments most often involve kinase inhibitors or monoclonal antibodies that target specific alterations known to drive the proliferation and survival of cancer cells (Fig. 1). These therapies have improved patient responses in many tumor types that previously had few effective treatments, such as RAF inhibitors for metastatic melanoma [1] and epidermal growth factor receptor (EGFR) inhibitors for EGFR mutant non-small cell lung cancer (NSCLC) [2].

**Fig. 2:**
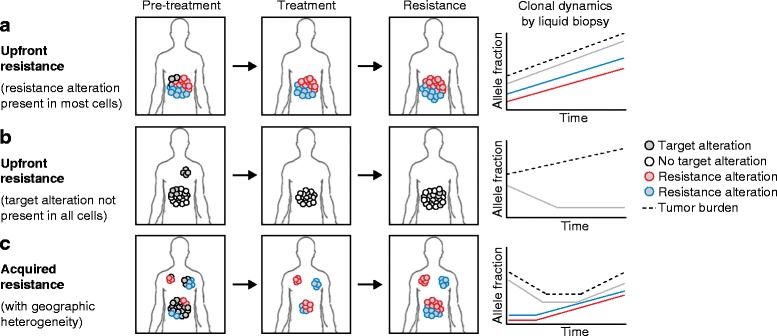
Heterogeneity and clinical resistance to targeted therapy. Genetic heterogeneity in human tumors can result in multiple outcomes for clinical responses to targeted therapy. In each case, monitoring tumor dynamics by analysis of liquid biopsies may improve clinical interventions. **a** A targetable genetic alteration (*gray*) may be present in most tumor cells, but may occur concurrently with resistance-driving mutations. This leads to upfront resistance despite the presence of the targetable alteration. **b** A targetable genetic alteration may only be present in a minority of tumor cells. In this case, the majority of cells in a particular tumor will exhibit upfront resistance. **c** Acquired resistance occurs when resistant subclones are selected from a heterogeneous tumor. Geographical resistance occurs when tumors are geographically heterogeneous and exhibit different genetic alterations at different tumor sites. In this case, each tumor will respond differently to targeted therapy

However, despite significant progress in strategies for cancer treatment using targeted therapies, resistance ultimately develops, resulting in disease progression in virtually every patient. This phenomenon also includes monoclonal antibodies used for immunotherapy, where recent studies have begun to characterize resistance mechanisms [3]. While the majority of cells in a tumor may contain a mutation that sensitizes them to a particular inhibitor, acquired resistance is thought to emerge due to tumor subclones harboring genetic differences that allow their survival and continued growth under drug pressure leading to resistant disease, as seen in Fig. 2 [4–6].

It is thought that acquired resistance is typically caused by the selection of small populations of tumor cells with pre-existing alterations that are capable of driving resistance (Fig. 2c). However, new research indicates a possible alternative model in which some drug-tolerant cells can remain static during treatment and spontaneously acquire de novo mutations over time that drive resistance [7]. Hata et al. [7] treated cells with EGFR inhibitor over long periods of time and separated populations with pre-existing, resistance-driving mutations from those able to persist in drug without growing. Drug-tolerant cells eventually appeared to acquire new mutations that led to resistance to EGFR inhibitor [7]. These data suggest that resistance may occur not only from the pre-existing heterogeneity of a patients’ disease, but that persistent, drug-tolerant cells can acquire new mutations as they adapt to certain treatments.

Next-generation sequencing studies of human tumors have increased our understanding of the vast heterogeneity of genetic alterations and resistance mechanisms in human cancer. Because of the heterogeneous nature of cancer cells, multiple resistance mechanisms may pre-exist in a given tumor, or between discrete tumors in a patient [8–10]. Analyses of tumor biopsies have revealed multiple resistance mechanisms in 50–80% of BRAF inhibitor-resistant melanoma patients [11, 12]. Two or more resistance mechanisms were also commonly detected within lung cancer [13–15] and colorectal cancer (CRC) patients [16, 17]. Importantly, studies have indicated that patients with high intratumoral heterogeneity have poorer survival or decreased treatment responses [18, 19].

Our understanding of the tremendous molecular complexity of tumor heterogeneity has been catalyzed by recent advances in “liquid biopsy” approaches, analyzing circulating tumor DNA (ctDNA), which is shed into the bloodstream by tumor cells throughout the body. Thus, ctDNA analysis provides a means to detect genomic alterations present in distinct tumor subclones in different metastatic lesions within an individual patient, thereby more effectively capturing the degree of tumor heterogeneity relative to a single-lesion tumor biopsy. Furthermore, liquid biopsy requires only a simple, non-invasive peripheral blood draw, allowing more frequent sampling of the tumor genome than standard tumor biopsy might allow [20].

Despite the large and increasing number of resistance mechanisms to targeted treatments, many converge on reactivation of the driving pathway. In *BRAF* mutant melanomas, for example, only 11% of detected resistance mutations were outside the mitogen-activated protein kinase (MAPK) pathway [11]. Detecting and identifying these drug-resistance mechanisms remains important for informing future treatment strategies to overcome resistance or delay disease progression.

In this review, we discuss studies revealing multiple, often convergent, resistance mechanisms to targeted inhibitors, mainly kinase inhibitors, or combination therapies, including studies using liquid biopsy approaches to assess resistance. We also consider future therapeutic options for resistant disease.

## Resistance to targeted therapies

Tumors develop resistance to all types of targeted therapy, including monoclonal antibodies and kinase inhibitors. The mechanisms by which tumors develop acquired resistance to therapy can typically be categorized into several classes, which include: (1) secondary alterations in the drug target, (2) activation of bypass signaling pathways, (3) adaptive or cell fate changes, and, more recently, (4) immune evasion.

One of the most straightforward ways in which a tumor can develop acquired resistance to a targeted therapy is through a secondary alteration (for example, mutation or amplification) to the drug target itself. An example is the common *EGFR* T790M “gatekeeper” mutation, which occurs after first generation anti-EGFR therapy in lung cancer and hinders drug binding [21]. Gatekeeper mutations occur in residues crucial for drug binding, allowing the target molecule to evade inhibition. Also, a recent study of acquired resistance to an inhibitor of the serine/threonine kinase mTOR revealed that some resistant cells developed activating mutations in the *mTOR* gene [22]. These mutants displayed higher levels of kinase activity than wild-type mTOR and were capable of driving resistance to mTOR inhibitors. The same *mTOR* mutations were also detected in some drug-naïve patients. Clearly, tumor cells possessing these alterations would be inherently resistant to these types of mTOR inhibitors, despite exhibiting high mTOR pathway activity [22].

Another common resistance mechanism involves activation of signaling pathways that “bypass” the drug target to maintain survival and proliferation. For example, *BRAF* mutant melanomas are highly addicted to the MAPK pathway. While BRAF inhibition initially results in responses, resistance ultimately develops, frequently via an alteration that reactivates the MAPK pathway [23], such as mutation or amplification of other MAPK pathway components. As a result, several clinical trials have been initiated for combination therapies that inhibit multiple nodes of the same pathway. Unfortunately, resistance also develops to these combination therapies, for example, those that inhibit MAPK in *BRAF* mutant melanoma and CRC [24–26]. In these cases, genomic alterations reactivate the MAPK pathway despite the presence of multiple inhibitors. Resistance mechanisms include *RAS* amplification or mutation, *BRAF* amplification or alternative splicing, and activating *MEK* mutations [24–26].

While resistance commonly occurs through reactivation of the original target pathway, resistance can also be driven by alterations activating parallel or redundant signaling pathways that can bypass the effects of a targeted inhibitor. The *EGFR* T790M mutation is a common cause of resistance to first generation EGFR inhibitors [21]. A third generation EGFR inhibitor, WZ4002, overcomes the *EGFR* T790M mutation [27, 28]. However, resistance ultimately develops to WZ4002 through EGFR-independent reactivation of the MAPK pathway [29, 30]. Tricker et al. [31] demonstrated that a combination of WZ4002 and the MEK inhibitor trametinib blocks MAPK reactivation and delays the acquired resistance that typically develops to EGFR inhibitors. However, unlike other models of resistance that develop to combination therapies through reactivation of the MAPK pathway, resistance to the combination of MEK inhibition and WZ4002 arises through activation of AKT/mTOR [31]. This suggests that despite complete inhibition of driver pathways, some cell populations may still adapt their signaling programs to escape targeting.

Therapeutic resistance can also arise through adaptive non-genetic mechanisms such as feedback reactivation of targeted pathways. Adaptive resistance can also occur through activation of parallel signaling pathways or the loss of negative feedback sources [32–34]. In *BRAF* mutant CRC, for example, RAF inhibitor treatment reduces the negative feedback signals that typically regulate the MAPK pathway. This loss in negative signal allows MAPK pathway reactivation, which in many cases is EGFR-dependent [32].

In addition to adaptive signaling, adaptive changes in differentiation status and cell fate are widely associated with resistance in cancer cells, although it remains unclear whether epithelial-to-mesenchymal transition is a driver of resistance [35–37]. One interesting example characterized a subset of NSCLC patients initially treated with EGFR inhibitor [38]. After an initial response, resistant disease developed that displayed classic characteristics of small-cell lung cancer. The transformation from lung adenocarcinomas to small-cell lung cancer was marked by loss of the *RB* gene, decreased EGFR expression, and increased neuroendocrine marker expression [38]—all typical of small cell lung cancers. As in this case, changes in differentiation status or transformation to a different tumor subtype are non-genetic resistance mechanisms that may emerge.

Finally, with the advent of effective immune-based therapies for various cancers, immune evasion is emerging as an important mechanism of acquired resistance. PD-1-based immunotherapy has provided durable, objective responses in a third of melanomas, with 75% of these responses lasting for at least 21 months [39]. However, like other targeted therapies, clinical resistance can arise through the selection of resistance-driving mutations during therapy. Recently, different mechanisms of immune evasion were characterized as drivers of resistance to PD-1 immunotherapy. In a study of paired biopsies from four patients with acquired resistance to anti-PD-1 therapy, one patient possessed a truncating mutation in the beta-2-microglobulin (*B2M*) gene [40]. This and other studies have detected such alterations in the *B2M* gene, which lead to loss of proper folding and localization of major histocompatibility complex class I, and immunotherapy resistance [40–42]. Additionally, two patients in the study had inactivating mutations in *JAK1* or *JAK2* with loss of heterozygosity. Exome sequencing, Sanger sequencing, and targeted resequencing of these sites in the baseline patient samples did not reveal these mutations at any detectable frequency, yet upon relapse the tumors were relatively homogenous. This suggests that the *JAK* mutations were present prior to treatment and were clonally selected. Functional analysis of the *JAK2* mutant revealed a complete loss of response to interferon gamma, while the *JAK1* mutant exhibited resistance to interferons alpha, beta, and gamma, effectively blocking interferon-induced growth arrest [40].

Previous studies have found that resistance to kinase inhibitors can contribute to changes in immune phenotype, leading to questions concerning the combination of kinase inhibitors and immunotherapies [43, 44]. Indeed, broader discussions of immunotherapy resistance and combination treatments with immunotherapy are presented elsewhere [3, 45].

## Geographic heterogeneity

Metastasis is a multi-step process that requires the selection of cell subpopulations capable of surviving in the circulation and creating a new metastatic colony. As a result of this strong selection, tumor cells occupying metastatic sites may possess a different genetic landscape to the primary tumor, or to other metastases in the same patient, resulting in tumor heterogeneity that varies by geographical distribution. Therefore, molecular testing of a biopsy from one metastatic site might not accurately reflect the mutational profile of the primary tumor or other metastases [46]. This type of geographic heterogeneity has been demonstrated in brain metastases which have been found to have divergent evolution from the primary tumor site. In half of these cases, alterations found in the brain metastases were potentially clinically actionable and were not detected in the primary tumor [47]. Accordingly, treatment selection based on the molecular profile of a single biopsy may yield resistance through a mixed response of different tumor lesions to treatment that can markedly alter clinical outcomes, as displayed in Fig. 2c. In patients, this phenomenon can drive either upfront or acquired resistance to agents used in the clinic.

We recently reported [48] an example of tumor heterogeneity driving upfront resistance in two esophagogastric cancer patients, in which biopsy of one metastatic site revealed high-level *MET* amplification. As a result of this finding, each patient was treated with a MET inhibitor and experienced a dramatic response in their metastatic disease. However, the primary disease continued to progress, leading to treatment failure (Fig. 2b). Analysis of biopsy samples from the primary tumor obtained before treatment, but never subjected to molecular analysis, revealed that *MET* amplification was not detected in the primary tumor, indicating that amplification either occurred spontaneously in a metastatic cell or was specifically present in a subpopulation of cells selected for upon the metastatic process [48].

Similarly, we reported a striking example of how tumor heterogeneity between individual metastases at the time of acquired resistance can lead to mixed response and treatment failure in a CRC patient following acquired resistance to the anti-EGFR antibody cetuximab [17]. Biopsy of a progressing liver metastasis revealed emergence of a *MEK1* K57T mutation. This mutation occurs downstream of EGFR and, therefore, was found to promote resistance to cetuximab in CRC cells, but this resistance could be overcome by combined treatment with an anti-EGFR antibody and a MEK inhibitor. The patient treated with this combination experienced a reduction in the size of the liver lesion containing the *MEK1* K57T mutation. However, other liver lesions progressed during this therapy, and the patient failed therapy due to a mixed response. Interestingly, liquid biopsy analysis of ctDNA isolated from serial blood draws during therapy showed a decline in MEK1 K57T levels, but a previously undetected *KRAS* Q61H mutation was detected prior to treatment that increased in levels despite therapy. No *KRAS* Q61H mutation was detected in the original liver lesion biopsy, but it was later found to be present in a biopsy of a neighboring liver metastasis that progressed through therapy [17].

These studies demonstrate how geographical resistance due to tumor heterogeneity can yield mixed responses to treatment, and they emphasize a key limitation to the use of single biopsies to assess mutation status and to guide the selection of subsequent therapy [17, 47, 48]. However, these cases also illustrate how using the liquid biopsy approach to evaluate ctDNA from patient plasma can provide a more comprehensive view of the heterogeneity of resistance mechanisms present in an individual patient [17, 48].

## Liquid biopsies to assess patient disease

Detailed studies have demonstrated that single tumor biopsies contain a small proportion of the genetic alterations present in a given tumor [10], may represent only a fraction of the disease present in a patient, and can miss potential geographical heterogeneity. Isolating ctDNA, sometimes called cell-free DNA, from liquid biopsies has the potential to capture the molecular heterogeneity of a patient’s disease more effectively, and without the need for a tissue biopsy [49–53]. Analyzing ctDNA from patient plasma can provide a more representative sample of a patient’s disease than a single solid tumor biopsy (Fig. 2).

For example, serial plasma samples of NSCLC patients on first-line erlotinib treatment were assayed for ctDNA targeting *EGFR* exon 19 deletions, *EGFR* L858R, and *EGFR* T790M [54, 55]. During periods of response to erlotinib, reduced levels of *EGFR* exon 19 deletions were detected. Additionally, resistance mutations in *EGFR* were detectable 4–24 weeks prior to radiographic progression [55], providing an earlier opportunity to intervene with next-line therapy. Similarly, digital droplet PCR was also used to evaluate *EGFR* dynamics during treatment with rociletinib, a third generation EGFR inhibitor [14]. In some patients, rociletinib resistance correlated with an increase in both the *EGFR*-activating mutation and T790M. Interestingly, levels of the *EGFR*-activating mutation increased in other patients with no change in T790M, indicating that increased T790 wild-type *EGFR* was the resistance mechanism [14]. In addition to the value of monitoring response and progression, ctDNA can be analyzed in cases where a solid tissue biopsy may not be possible [54].

Analysis of ctDNA is leading to a broader view of tumor heterogeneity, as a greater representation of a patient’s disease can be assessed in a plasma sample, particularly when coupled with next-generation sequencing strategies. In one example, ctDNA was isolated from serial plasma samples taken from NSCLC patients on a clinical trial for a third generation EGFR inhibitor and analyzed with a targeted cancer personalized profiling by deep sequencing (CAPP-Seq) panel. Most of these patients had already been treated with at least one EGFR inhibitor and had progressive disease during this treatment. Previous studies of tumor biopsies found that a minority of patients (5–15%) exhibited multiple mechanisms of EGFR inhibitor resistance. However, CAPP-Seq analysis of ctDNA revealed that almost half of resistant *EGFR* T790M patients exhibited multiple resistance mechanisms, including *MET* amplification, *ERBB2* amplification, or additional mutations in *EGFR*, *RB1*, or *PIK3CA*. These data demonstrate that solid tumor biopsies are likely to under-represent the number of genomic alterations present in a patient, and this may have important implications for outcomes in response to newer EGFR inhibitors. For example, detection of *MET* amplification in response to the third generation EGFR inhibitor rociletinib implicates the addition of a MET inhibitor as a reasonable next-line therapy [15].

In addition to providing a broader genetic snapshot of a patient’s disease, other benefits of liquid biopsies include that they can be non-invasively performed at any time during treatment. Solid tumor biopsies are often invasive, so their number is limited to avoid unnecessary risk and inconvenience to the patient. Also, unlike solid tumor biopsies, liquid biopsies can continue to be performed when tumors are below radiographic detection. Drawbacks of using ctDNA from liquid biopsies to monitor resistance are largely related to sensitivity issues from low DNA yields. Low ctDNA levels may limit the ability to analyze a sample by high-throughput approaches, while also increasing the frequency of potential false positives or negatives. Also, ctDNA isolation does not allow studies that require intact cells. These analyses, such as histological staining or in situ hybridization, are possible with circulating tumor cells or solid tumor biopsy. These experiments may be important in determining whether specific genetic alterations occur in the same cells or in separate tumor subpopulations. Thus, liquid biopsy may complement standard analyses of solid tumor biopsies, and integrating these two approaches may be an important approach to guide clinical decision-making. Further studies will determine the efficacy of these approaches in multiple tumor types and contexts.

## Convergent mechanisms of resistance

The pronounced heterogeneity of resistance mechanisms observed between patients, and between different tumor subclones in the same patient, presents a daunting obstacle to the development of combination therapies, or second- and third-line inhibitors, intended to overcome resistance [25, 56–58]. In many cases, however, multiple resistance mechanisms often converge to reactivate the original signaling pathway to which resistant tumor cells remain addicted. This convergence upon a common signaling node offers an attractive opportunity to overcome heterogeneous resistance mechanisms by therapeutically targeting a single pathway (Fig. 1).

For example, CRCs resistant to anti-EGFR antibodies frequently develop acquired resistance through *KRAS*, *NRAS*, and *BRAF* mutations. These mutations each converge upon reactivation of the MAPK pathway, and cells remain sensitive to inhibition of MEK in combination with EGFR [16]. Studies of resistance to combination therapies have also revealed strong addiction to the originally targeted pathway, indicating that further inhibition may restore tumor sensitivity.

Indeed, vertical inhibition of the MAPK pathway—with BRAF inhibitors in combination with MEK and/or EGFR inhibitors—provides improved responses in *BRAF* mutant CRC compared to BRAF inhibitor alone [59, 60]. However, even if a patient initially responds to a combination of inhibitors targeting two or three nodes of the MAPK pathway, resistance ultimately develops. Through analysis of biopsies taken before and after treatment, as well as the generation of resistant cell lines in culture, we have learned that the vast majority of resistance mechanisms to combination therapy ultimately reactivate the MAPK pathway [25, 61]. Mechanisms include *KRAS* mutation and amplification, as well as amplification of mutant *BRAF*, and *MEK* mutations [25, 61], which all converge to reactivate extracellular signal-regulated kinase (ERK) in tumor cells. Remarkably, resistant CRCs harboring any of the above alterations retain sensitivity to direct ERK inhibition or ERK-based combinations, illustrating how targeting a common convergent signaling node can potentially overcome multiple resistance mechanisms [25, 61].

Convergent mechanisms of resistance to targeted therapy can occur within a single patient. For example, analysis of five vemurafenib-resistant metastases from a single patient with *BRAF*-mutant melanoma revealed that MAPK signaling was reactivated in each of the five tumors, albeit through discrete mechanisms. Mutant *BRAF* was amplified in three lesions; one lesion contained a *BRAF* fusion and another contained an activating insertion in the *MEK1* gene [23]. This heterogeneity of resistance mechanisms within one patient illustrates the challenge in treating resistant tumors driven by multiple mechanisms. However, as each of these resistance mechanisms has converged on reactivation of the MAPK pathway and increased phosphorylated ERK levels, improved MAPK targeting through combination therapies or direct ERK targeting remains a treatment option for this type of resistance [62].

Similarly, a patient with metastatic breast cancer with an activating *PIK3CA* mutation was treated with the PI3Kα inhibitor BYL719 and eventually developed BYL719 resistance [63]. Analyses of multiple metastases from the patient revealed that each metastatic lesion harbored different genetic alterations that resulted in *PTEN* loss, the source of BYL719 resistance [63]. While each tumor site likely arose from different tumor subclones containing different *PTEN* alterations, this mechanism of convergent evolution was a common source of resistance across multiple tumor sites. These cells with *PTEN* loss were then sensitive to PI3K p110β inhibition [63].

In *ALK*-rearranged NSCLC resistant to crizotinib (an ALK/MET inhibitor), only 31% of patients exhibited *ALK* mutations or *ALK* amplification post-treatment [64]. Following treatment with second-generation ALK inhibitors, it was found that *ALK* mutations were more likely to drive resistance, occurring at a rate of 54, and 12.5% of these patients contained multiple *ALK* mutations. These data suggest that while other resistance mechanisms can exist, subclonal selection by ALK inhibitor treatment results in the progressively increased likelihood that on-target resistance (that is, resistance to the originally targeted protein, here ALK) will arise. Treatment with the third generation ALK inhibitor lorlatinib has been most successful against tumors with *ALK* mutations that arose during therapy with second-generation ALK inhibitors [64], providing a prognostic indicator for lorlatinib treatment in ALK-addicted progressive disease.

## Clinical approaches for heterogeneous tumors

With the increase in awareness and detection of tumor heterogeneity and multiple genetic sources of resistance, our focus now turns to what clinical approaches can be taken for optimal benefit. Some combination therapies seek to overcome sources of adaptive resistance by targeting multiple signaling nodes. In other cases, new inhibitors are in development that target known routes of resistance, and these may be useful when combined with currently used inhibitors to prevent outgrowth of known resistance mutations.

### Combination therapies targeting convergent mechanisms of resistance

While studying individual resistance mechanisms is valuable for informing future treatment approaches, specifically targeting individual resistance alterations as they arise is unlikely to be clinically feasible. As described above, however, many genetic resistance mechanisms converge on reactivation of the intended protein or pathway target, as in the case of the MAPK pathway in *BRAF* mutant melanoma or CRC [11, 25]. In this scenario, using recently developed inhibitors to target ERK as a common convergent signaling node allows multiple resistance mechanisms to be simultaneously overcome. Finding new approaches to maintaining the inactivation of key signaling pathways and “escape routes” is essential to overcoming and delaying resistance.

One example is in *ALK*-rearranged NSCLC, in which a patient exhibited acquired resistance to crizotinib caused by a mutation in *ALK* [65]. This patient’s disease responded to the third generation ALK inhibitor lorlatinib, yet subsequently developed resistance after the acquisition of a second *ALK* mutation. Interestingly, the second acquired mutation resensitized ALK to crizotinib, allowing the patient to respond to this inhibitor a second time [65]. This unique example of resensitization to a compound emphasizes that addiction to a key tumor driver is the likely driver of resistance, and overcoming this will most often focus on blocking reactivation of the same pathway. Like ALK, second and third generation inhibitors are being developed for several targets, notably EGFR and mTOR, which can extend treatment options for kinase-driven cancers as they progress [22, 64].

In cases where resistance develops during treatment with an inhibitor of receptor tyrosine kinases (RTKs) such as EGFR, or ALK, common resistance mechanisms include on-target mutations [13, 14, 51, 65]. This phenomenon is similar in the cases of resistance to monoclonal antibodies targeting RTKs, such as cetuximab or panitumumab for EGFR. To block developing resistance, a compound mixture has been developed that contains two or three non-overlapping antibodies targeting EGFR [66, 67]. In CRCs that were resistant to cetuximab because of an acquired *EGFR* mutation, resistance was overcome by using the antibody mixtures Sym004 or MM-151 [66, 67]. Additional antibody or inhibitor mixtures will likely be developed in order to overcome resistance to first-line treatments and delay the development of additional resistance.

### New compounds targeting key signaling nodes

In addition to next-generation inhibitors, new compounds are being developed that may prevent resistance by targeting key pathway nodes that are known to be crucial for driving resistance. One example is the development of inhibitors that directly target ERK activity, including ulixertinib and SCH772984 [68, 69]. Since many common resistance mechanisms result in MAPK pathway reactivation [11, 25, 31, 70], using an ERK inhibitor in combination with other MAPK pathway inhibitors may provide improved responses. Indeed, ERK inhibition was able to overcome resistance to vertical inhibition of MAPK pathway components in *BRAF* mutant CRC and melanoma [25, 61, 70–72]. Additionally, where *KRAS* mutant cells are intrinsically resistant to MAPK inhibitors that target RAF and MEK, because of adaptive reactivation of P-ERK, direct inhibition of ERK may enhance responses in these cell types [73, 74]. MEK inhibitors are also currently being investigated for many combination treatments. Adding ERK inhibitors, or substituting ERK for MEK, may improve MAPK pathway targeting in many cases.

Several compounds target BRAF, yet unfortunately they have little affinity for other RAF isoforms. In *BRAF* wild-type cells, BRAF inhibitors induce dimerization of RAF proteins, leading to paradoxical activation of the RAF-MEK-ERK cascade [75, 76]. This activation is a major limitation for the clinical use of RAF inhibitors and also results in side effects in the skin for those with *BRAF* mutant tumors [1]. LY3009120 is a new RAF inhibitor with approximately equal affinity for all RAF isoforms. This compound induces dimerization of RAF, yet blocks kinase activity of the dimers in *RAS* and *RAF* mutant cells [77]. Limiting the paradoxical reactivation in *BRAF* wild-type cancers, as well as potentially limiting the side effects in skin of BRAF inhibitors, may provide therapeutic benefit for many patients. In vemurafenib-resistant melanoma, LY3009120 was able to block RAF activity, despite the presence of several MAPK-reactivating mechanisms [77]. Indeed, pan-RAF inhibition in combination with MEK inhibition can overcome intrinsic resistance to MAPK inhibition in *RAS*-mutant cancers, as well as acquired resistance in *RAS* and *RAF*-mutant cancers [72, 78–80].

RAS activity is required for the downstream signaling of many RTKs. It has been found that RAS activity is dependent on dephosphorylation of Tyrosine32 (Y32), which results in RAS binding to RAF and its GTPase-activating protein. The dephosphorylation of Y32 is mediated by the phosphatase SHP2, which directly activates RAS downstream of RTK signaling [81]. This implicated the SHP2 phosphatase as a therapeutic target for RTK-driven cancers, leading to the development of a SHP2 inhibitor, despite the challenges of creating phosphatase inhibitors [82]. Indeed, SHP2 inhibition reduced P-ERK levels in RTK-addicted cell lines, and accordingly reduced their survival and proliferation. Additionally, the compound successfully induced tumor regressions in a xenograft model. As might be expected, SHP2 inhibition had no effect in *KRAS* or *BRAF* mutant cancers [83]. It remains to be seen whether the SHP2 inhibitor can cooperate with other inhibitors to reduce the adaptive feedback that occurs through RTK signaling in response to inhibition of several major pathway nodes, such as with MAPK inhibition in *KRAS* mutant cancers.

### Combination and sequential targeted therapy

The clinical problem of heterogeneity can be approached with multiple treatment strategies. The first involves combination therapies that target known mechanisms of adaptive or acquired resistance that can emerge during treatment. This approach delays the progression of disease by preventing outgrowth of the most common resistant clones. The second approach involves monitoring the emergence of resistance-causing alterations in a patient’s disease by liquid biopsy. Treatments can be adjusted based on the findings of this testing to sequentially target emerging resistance mechanisms.

Using combination therapies to address upfront resistance mechanisms has the benefit of targeting a greater percentage of cancer cells than sequential targeting. Additionally, several compounds exhibit synergistic effects that result in improved pathway targeting. Modeling of tumor evolution has demonstrated greater benefits with combinatorial therapy versus sequential therapy because of the potential for drug synergy and the potential for particular alterations to cause cross-resistance to multiple compounds [84]. For example, when lung cancers with the *EML4-ALK* fusion were treated with ALK inhibitor alone, either adaptive signaling or acquired mutations resulted in reactivation of the MAPK pathway. When a MEK inhibitor was combined with the ALK inhibitor, cells exhibited stronger and longer-lasting responses [85]. Here, sequential therapy would not be beneficial, since either monotherapy alone would be ineffective.

Similarly, EGFR antibody treatment in CRC results in MAPK pathway reactivation. Resistant cells were found to contain alterations to *RAS* and *RAF* genes, leading to permanent pathway activation. Combination treatment with agents targeting EGFR and MEK impaired growth of these resistant cells, and—importantly—a xenotransplant from a patient who acquired EGFR antibody resistance responded to the combined targeting of EGFR and MEK [16]. Additionally, combination treatment targeting EGFR and MEK was able to block the outgrowth of resistant clones, indicating that this combination can prevent resistance in addition to overcoming acquired resistance [86].

**Fig. 3:**
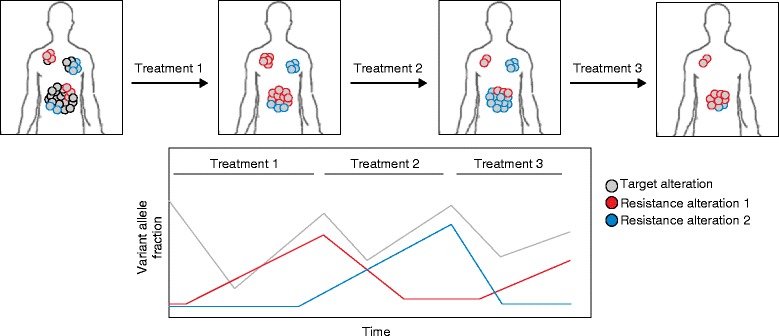
Sequential targeted therapy assessed by longitudinal liquid biopsy. At the beginning of targeted therapy, all cells in the patient’s tumor possess an actionable genetic alteration (*gray*). The first treatment administered targets this first alteration. Liquid biopsy analysis demonstrates an initial decrease in the target alteration during treatment 1, yet reveals the outgrowth of an alteration causing resistance to treatment 1 (*red*). The red subclone can be targeted with treatment 2, where liquid biopsy analysis reveals a decrease in the frequency of resistance alteration 1. During this time, however, a third genetic alteration (*blue*) increases in frequency. This third mutation is resistant to treatment 2, yet is sensitive to treatment 3. During treatment 3, the frequency of the blue clone decreases, while residual clones harboring the first resistance mutation (*red*) may persist

However, using multiple targeted agents in combination may not be tolerable, and toxicity is likely to limit the efficacy and feasibility of this approach in the clinic. Accordingly, an alternative strategy would be to carry out sequential therapies directed against specific resistant subclones, using real-time liquid biopsy ctDNA analysis to monitor clonal evolution and guide adaptation of therapy (Fig. 3). Sequential therapies have some benefits over combination therapy, including the ability to use optimal doses without the need to reduce doses due to toxicity concerns. Similarly, some patients may have conditions that prevent them from tolerating some therapy combinations simultaneously. As liquid biopsy technologies become more commonly used in the clinic, treatment regimens may be altered to adjust to the molecular changes in a patient’s overall tumor burden more rapidly, as resistance mechanisms emerge, prior to the radiographic detection of resistant disease. Furthermore, recent studies have suggested that some resistant subclones that emerge during therapy may decrease in prevalence after the therapy is discontinued, and that this can be monitored in ctDNA, suggesting that a patient may later be successfully re-challenged with the same therapy [87].

Commonly, patients with *EGFR* mutant lung cancers become resistant to first-line EGFR-targeted therapies due to the acquisition of a gatekeeper mutation in *EGFR*, T790M [21]. Patients with *EGFR* mutant lung cancers with the T790M mutation were enrolled in a trial of rociletinib, a third generation inhibitor that targets T790M-mutant EGFR [14]. During the study, solid tumor biopsies were collected from patients before the study and after acquiring resistance to rociletinib. In some of these patients, analysis of the tumor biopsies revealed an increased frequency of *EGFR* that is wild type for the T790 mutation as a mechanism of rociletinib resistance, as discussed earlier [14]. Notably, comparison of solid tumor and liquid biopsies from these patients revealed similar results in the ratios of wild type to mutant *EGFR* detected, indicating that liquid biopsies may be suitable for longitudinal assessment of a patient’s tumor [14]. These data suggest that monitoring via liquid biopsy can provide adequate information regarding the resistance mechanisms present in a tumor, and can inform subsequent treatment decisions without the need for a second solid tumor biopsy.

In a recent trial [88], the FGFR2 inhibitor BGJ398 was found to be effective for intrahepatic cholangiocarcinoma patients with activating *FGFR2* fusions; however, resistance developed after a short response period. Serial analysis of ctDNA from three patients who developed acquired resistance to therapy following initial clinical benefit revealed the emergence of polyclonal secondary mutations in the *FGFR2* kinase domain, which drive resistance to BGJ398 [89]. Mechanistic studies revealed that each of the multiple resistance mutations in *FGFR2* emerging in these patients was surmountable by structurally distinct FGFR inhibitors, but that no single inhibitor could effectively overcome them all. However, if a specific “next line” FGFR inhibitor could be selected based on the profile of emerging *FGFR2* mutations as detected by real-time liquid biopsy, this may allow clinical application of the most effective therapeutic strategy for each patient to prolong clinical benefit. This example suggests the potential for longitudinal monitoring of emerging resistance alterations to inform adaptation of subsequent treatment strategies for patients with resistant disease.

## Conclusions

Next-generation sequencing of patient biopsies has revealed that tumors contain vastly heterogeneous genetic alterations in multiple subclones. This heterogeneity in patient tumors provides the fuel for upfront and acquired resistance to targeted therapies. The stage in tumor development at which the resistance mutation occurs dictates the clinical presentation of resistance, such as upfront resistance, acquired resistance at the primary site, or acquired resistance at a metastatic site (Fig. 2). As there is the potential for multiple resistance mechanisms within a single patient, particularly between multiple lesions in a patient, analysis of liquid biopsies can achieve a more accurate representation of resistance. These technologies provide an exciting opportunity to more closely monitor the emergence of new genetic alterations without a solid tumor biopsy, and may lead to more rapid adaptation of sequential therapies to overcome specific resistance mechanisms detected in the blood. Despite the vast heterogeneity of resistance-driving mechanisms, many of these mechanisms converge on reactivation of the same protein or pathway. Targeting crucial nodes required for reactivation of these key pathways provides a therapeutic opportunity for resistant cancers, despite the presence of multiple resistance mechanisms. While resistance may never be entirely prevented, the development of new inhibitors and combination approaches may help to treat common drivers of resistance or delay progressive disease.

## Abbreviations

CRC: Colorectal cancer
NSCLC: Non-small cell lung carcinoma
RTK: Receptor tyrosine kinase
